# Supplementary material for: Reactive wetting enabled anchoring of non-wettable iron oxide in liquid metal for miniature soft robot
Source: Nat Commun. 2023 Oct 7;14:6276. doi: 10.1038/s41467-023-41920-4 (PMC10560245; doi:10.1038/s41467-023-41920-4)
Supplement: Supplementary file 1 — Supplementary Information [file 41467_2023_41920_MOESM1_ESM.pdf]

Supplementary Information

Reactive Wetting Enabled Anchoring of Non-wettable Iron Oxide in Liquid Metal for Miniature Soft Robot

Yifeng Shen[1], Dongdong Jin[1*], Mingming Fu[1], Sanhu Liu[2,3], Zhiwu Xu[2,3], Qinghua Cao[4], Bo Wang[4], Guoqiang Li[1], Wenjun Chen[1], Shaoqin Liu[5], Xing Ma[1,3,5*]

[1] Sauvage Laboratory for Smart Materials, School of Materials Science and Engineering, Harbin Institute of Technology (Shenzhen), Shenzhen 518055, China
[2] School of Materials Science and Engineering, Harbin Institute of Technology, Harbin 150001, China
[3] State Key Laboratory of Advanced Welding and Joining, Harbin Institute of Technology, Harbin 150001, China
[4] School of Materials Engineering, Shanghai University of Engineering Science, Shanghai 201620, China
[5] Key Laboratory of Microsystems and Microstructures Manufacturing, School of Medicine and Health, Harbin Institute of Technology, Harbin 150080, China
*Corresponding author.
Email: jindongdong@hit.edu.cn (D. Jin); maxing@hit.edu.cn (X. Ma)

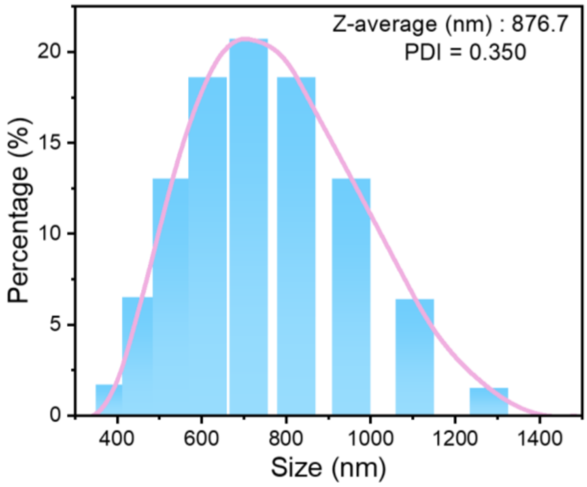

Fig. S1. Particle size distribution of Fe_3O_4 nanoparticles prepared by the hydrothermal method. Source data are provided as a Source Data file.

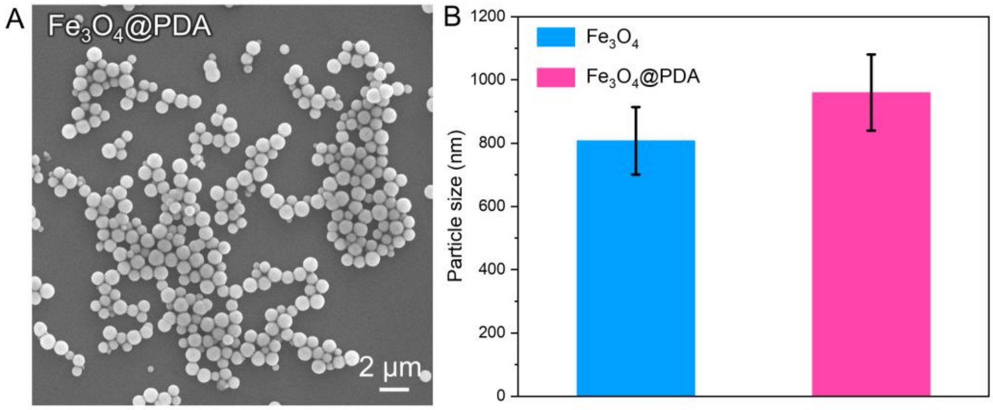

Fig. S2. Characterizations of Fe_3O_4@PDA nanoparticles. (**A**) SEM image showing fabricated Fe_3O_4@PDA nanoparticles. Each experiment was repeated independently for 3 times with similar results. (**B**) Size comparison between Fe_3O_4 and Fe_3O_4@PDA. The data are obtained by measuring the diameters of 100 nanoparticles in the SEM images, which derive the standard deviation (SD). Source data are provided as a Source Data file.

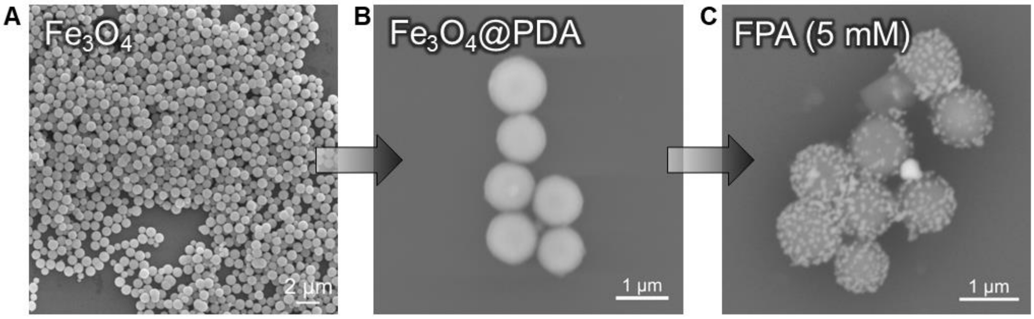

Fig. S3. SEM images of (**A**) bare Fe_3O_4 nanoparticles, (**B**) Fe_3O_4@PDA nanoparticles, and (**C**) Fe_3O_4@PDA@Ag (5 mM) nanoparticles. Each experiment was repeated independently for 3 times with similar results.

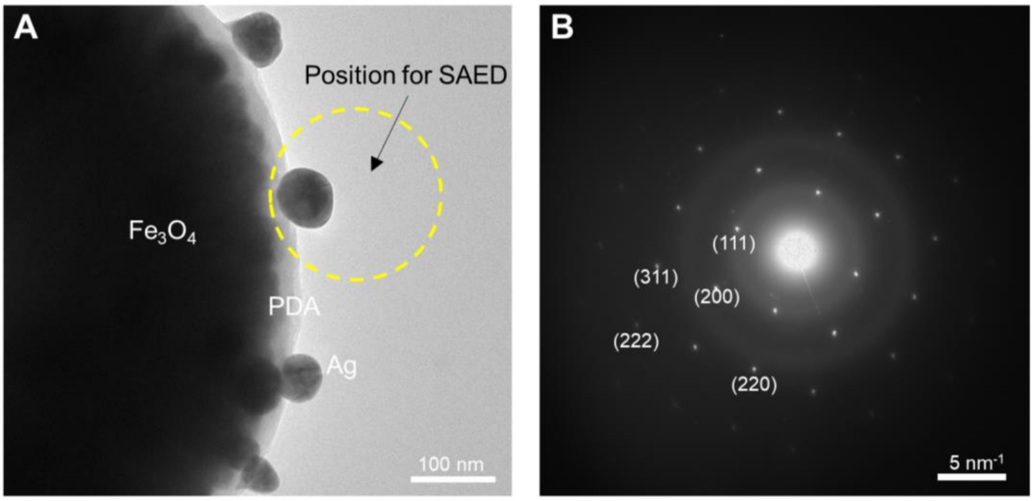

Fig. S4. Characterizations of Fe_3O_4@PDA@Ag nanoparticles. (**A**) TEM image of Fe_3O_4@PDA@Ag (5 mM). The thickness of PDA layer is identified to be 33.11 ± 3.60 nm (n = 10 independent particles). (**B**) Selected area electron diffraction (SAED) showing the characteristic crystal planes of decorated Ag nanoparticle.

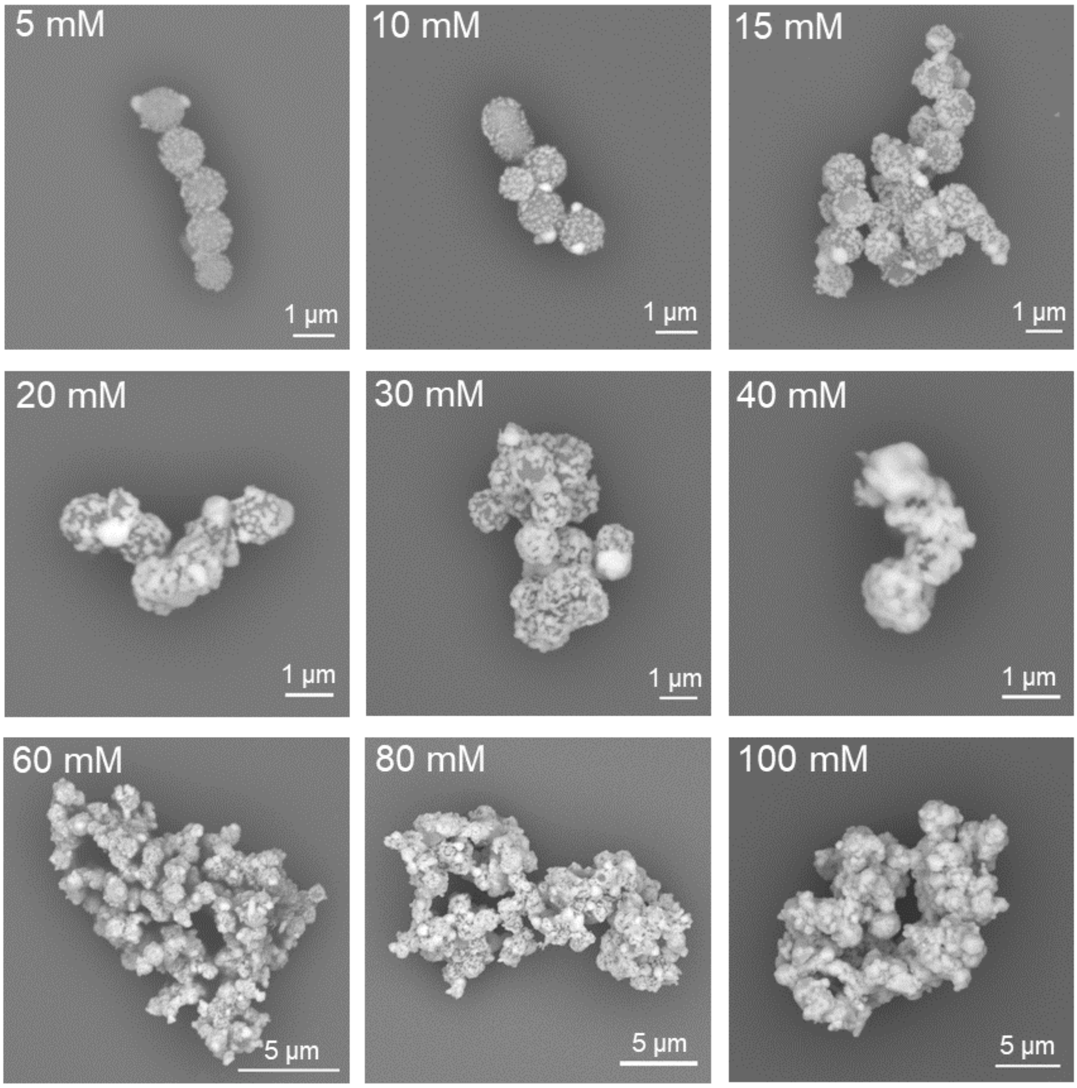

Fig. S5. SEM images showing various morphologies of Ag shells modified on the Fe_3O_4@PDA as the Ag^+ concentration increases. Ag nanoparticles stick and scatter on the surface of Fe_3O_4@PDA when Ag^+ concentration is 5 mM. Then the Ag shell is not continuous and thick until the concentration reaches 40 mM due to the Ostwald ripening of Ag nanoparticles. Once the concentration is beyond 40 mM, inner Fe_3O_4@PDA nanoparticles are covered entirely by thick Ag shell. Moreover, the monodispersity of magnetic particles will be weakened for further ripening and growth of sliver when the Ag^+concentration is over 60 mM. Each experiment was repeated independently for 3 times with similar results.

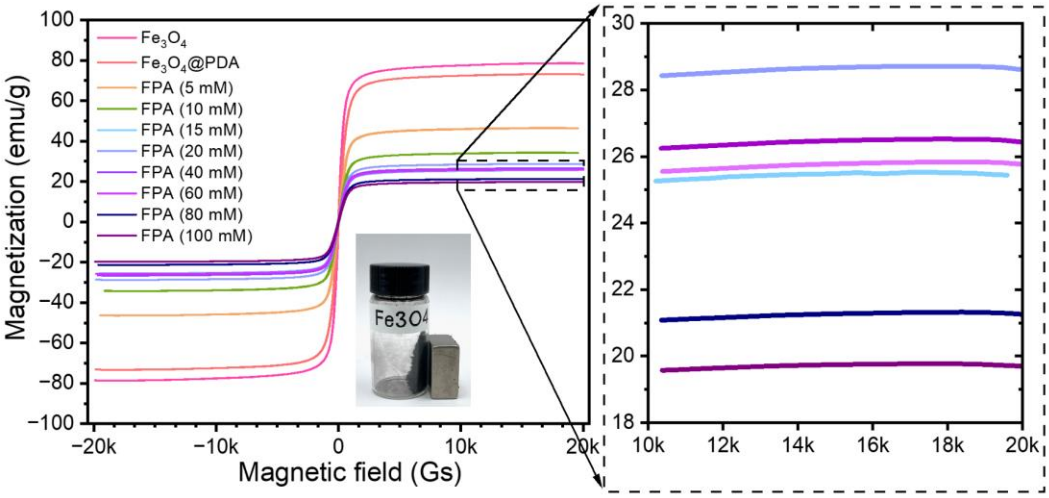

Fig. S6. Hysteresis loops at room temperature for different magnetic particles, with the inset showing the strong magnetic response of pure Fe_3O_4 powders. As PDA layers and Ag shells gradually functionalize the Fe_3O_4 nanoparticles, the saturated magnetizations per unit mass decline because of the lower mass fraction for Fe_3O_4 and the diamagnetism of Ag and PDA. Source data are provided as a Source Data file.

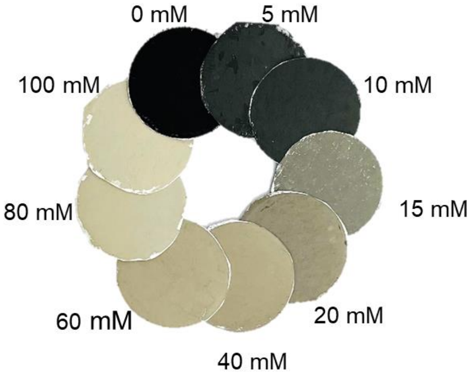

Fig. S7. Optical image exhibiting filter membranes spread with a various range of magnetic particles. With the increasing amount of Ag, the color of magnetic particles changes from black to silvery white. To acquire smooth filter membranes covered with magnetic particles, we use a double-roll machine to press these filter membranes after suction filtration.

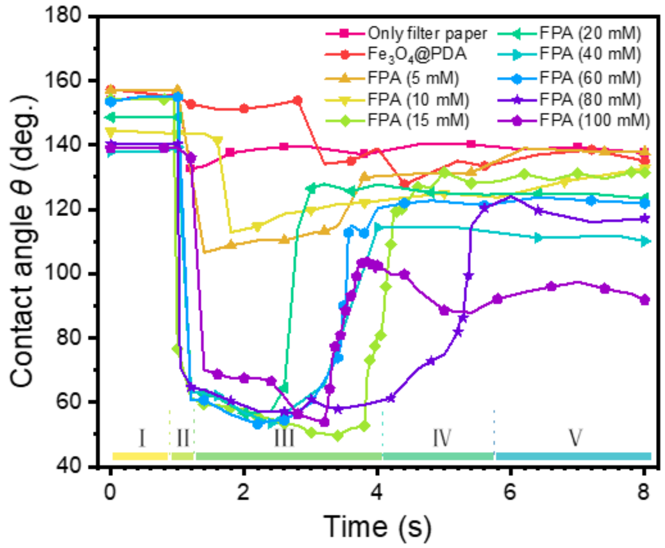

Fig. S8. Contact angles between EGaIn droplet and substrates with various magnetic powders as a function of time, where the dynamic process can be divided into five stages. Source data are provided as a Source Data file.

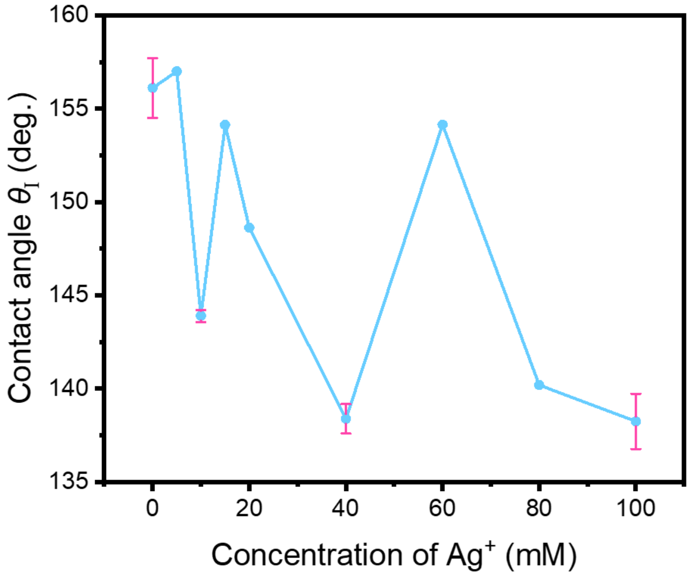

Fig. S9. The effect of Ag^+ concentration on contact angle during the first stage. All values represent the mean ± SD for n = 3 independent experiments. Source data are provided as a Source Data file.

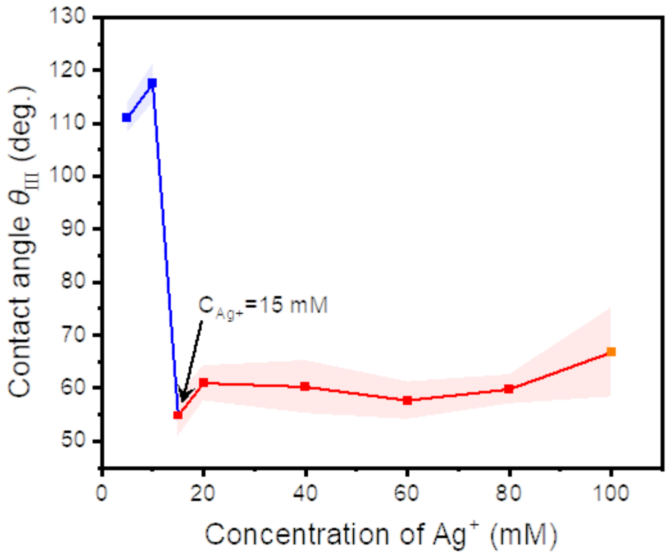

Fig. S10. The effect of Ag^{+} concentration on contact angle during the third stage. Source data are provided as a Source Data file.

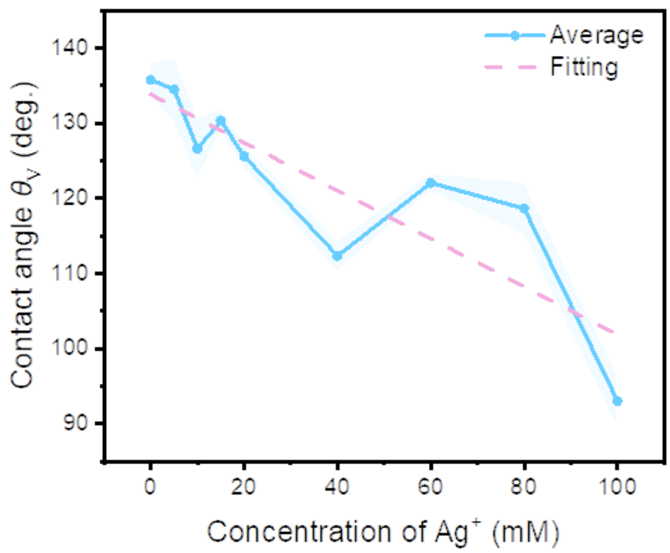

Fig. S11. The effect of Ag^+ concentration on contact angle during the final stage. Source data are provided as a Source Data file.

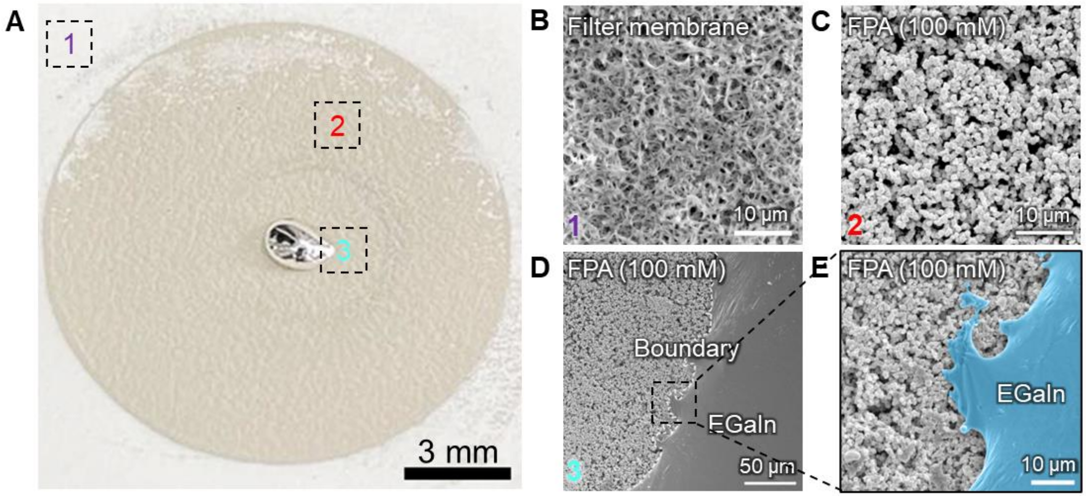

Fig. S12. Characterization for a filter membrane with FPA (100 mM) after contact angle test. (**A**) Optical image of a filter membrane with FPA (100 mM), on which an EGaIn droplet (~10 μL) is dropped. The EGaIn/FPA (100 mM) system is divided into three parts for further SEM imaging, where regions 1, 2, and 3 represent the filter membrane, FPA (100 mM) particles, and the boundary between EGaIn and FPA (100 mM), respectively. (**B**) SEM image of region 1, where the pore of filter membrane is measured to be the same as the nominal data (~0.45 μm). (**C**) SEM image of region 2, where dense FPA (100 mM) particles overspread the filter membrane substrate. (**D**) SEM image of region 3, where EGaIn wet with FPA (100 mM). (**E**) Zoom-in image of the boundary showing the wettability relationship between EGaIn and FPA (100 mM), where the blue area represents EGaIn.

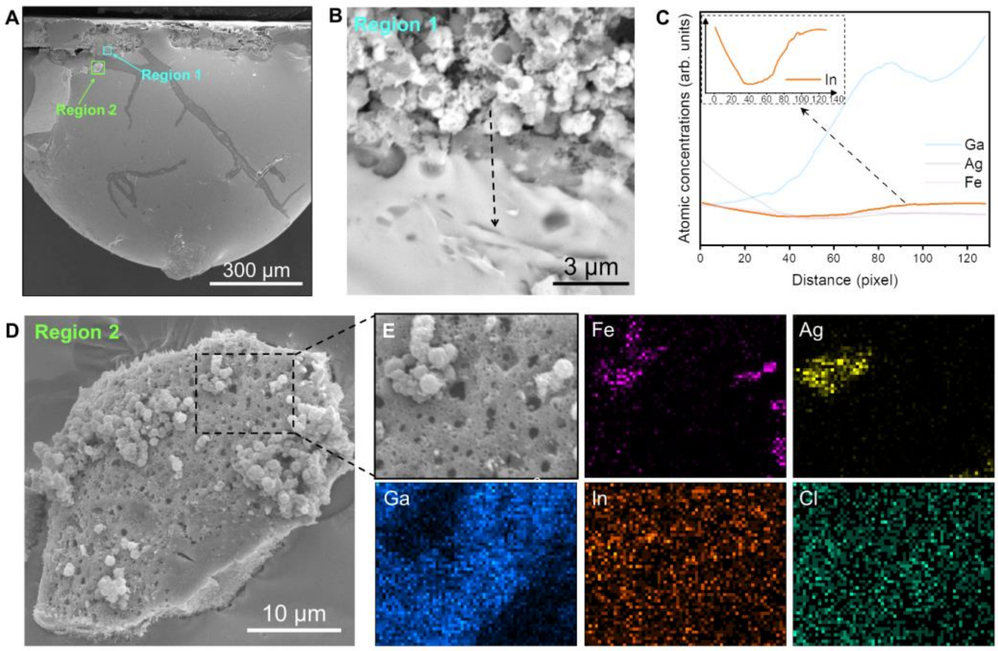

Fig. S13. Cross-sectional characterization for a filter membrane with FPA (100 mM) after contact angle test. (**A**) SEM image of the whole section of a filter membrane with FPA (100 mM) and EGaIn droplet. Two representative regions are further magnified. (**B**) SEM image of region 1 showing the interface between EGaIn and FPA (100 mM), where a black dotted line represents the trajectory of line scanning. (**C**) The plot of atomic concentrations for elements Ga, Ag, In, and Fe measured from every pixel of the trajectory upon line scanning. Atomic concentrations of element Fe and element Ag decrease while the atomic concentration of element Ga increases along this trajectory. As for the element In, we find that it appears in the area of FPA (100 mM), accompanied by reactive wetting during the process of liquid metal droplet contraction and extension. (**D**) SEM image of region 2 showing a porous structure with FPA nanoparticles. (**E**) Local magnification and mapping of the image in the black box of region 2 indicating that the main body of porous matter consists of crystalline chloride (maybe $GaCl_3$ and $InCl_3$). The hydrogen produced from the reaction between HCl and EGaIn or from the galvanic reaction between Ag and EGaIn continuously releases to promote the generation of these micropores [1-3]. Each experiment was repeated independently for 3 times with similar results. Source data are provided as a Source Data file.

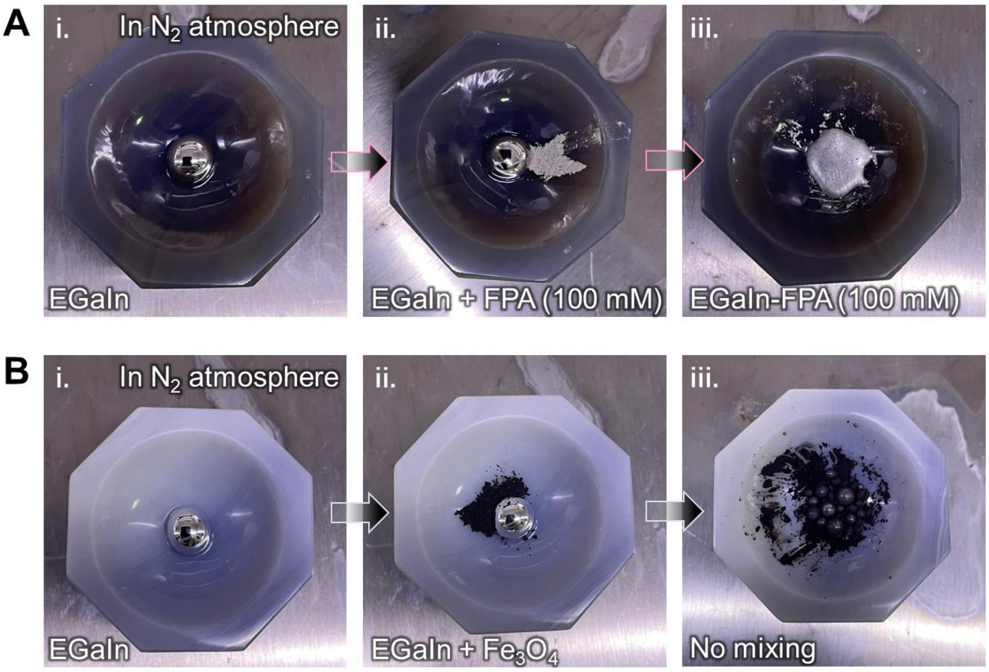

Fig. S14. Mechanical grinding of EGaIn liquid metal with Fe_3O_4@PDA@Ag (FPA (100 mM) (**A**) and Fe_3O_4 (**B**) powders in a glovebox filled with nitrogen gas.

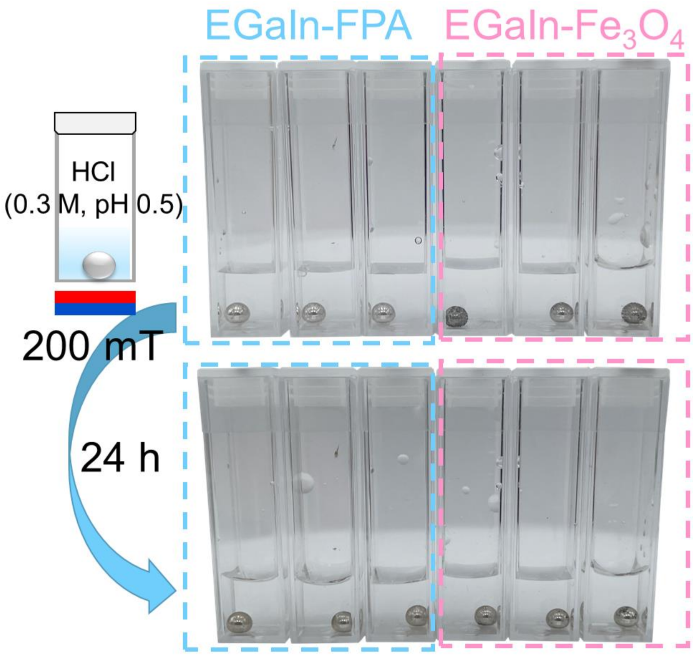

Fig. S15. Illustration and optical images of magnetic suspension stability tests for both EGaIn-5% FPA (100 mM) and EGaIn-1.25% Fe_3O_4 in acidic solution (0.3 M HCl solution).

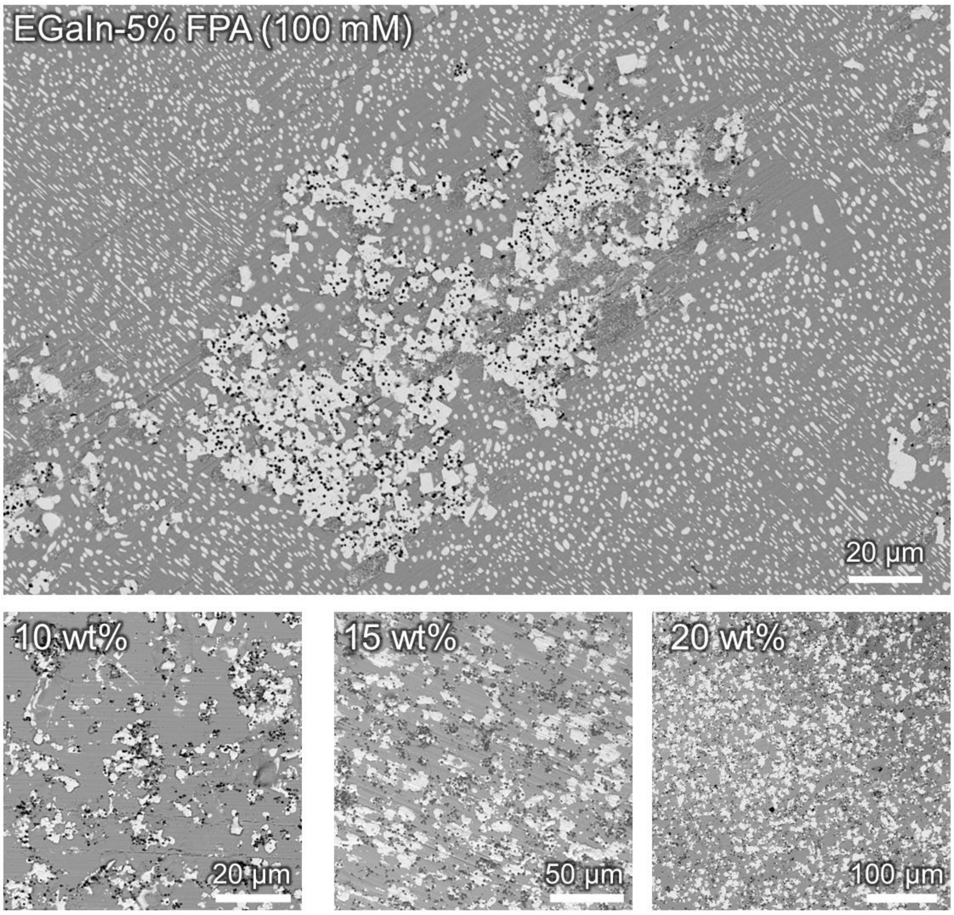

Fig. S16. SEM images of EGaIn-FPA (100 mM) with different FPA (100 mM) particle mass fractions. The results show that almost Fe_3O_4 nanoparticles are embedded in the Ag_xIn_y IMCs when the FPA (100 mM) mass ratio is relatively low (<10 wt%). Magnetic nanoparticles in low proportion may escape from the restriction of IMCs and distributed in the liquid metal matrix once the FPA (100 mM) mass ratio increases. The reasons can be concluded to explain the experimental phenomenon as follows. Firstly, the break of Ag_xIn_y IMCs upon mechnical stiring endows the Fe_3O_4 leakage. Next, the coordination binding between the empty electronic orbits of EGaIn and the lone electron pair of Fe_3O_4 helps these magnetic nanoparticles to mix with EGaIn [4]. Finally, oxide films warp Fe_3O_4 to improve the wettability with liquid metals upon mechanical grinding [5]. Each experiment was repeated independently for 3 times with similar results.

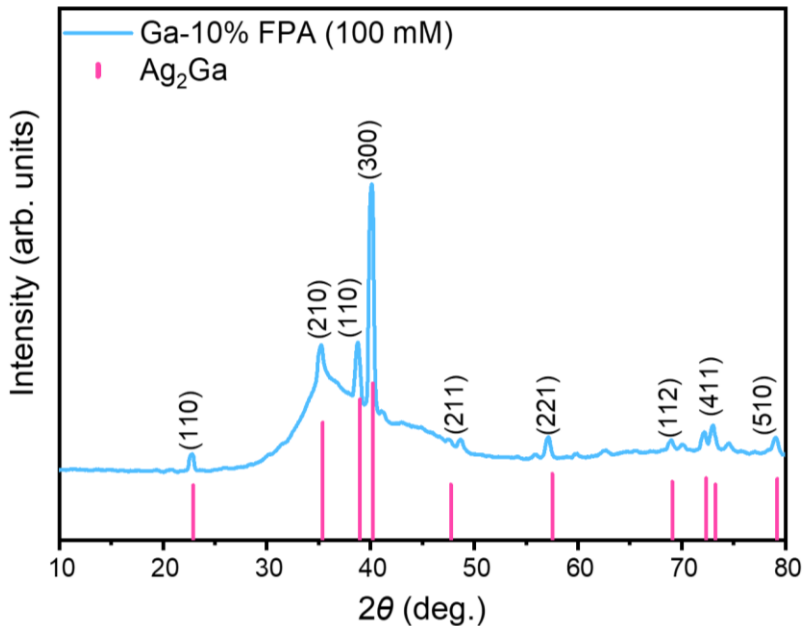

Fig. S17. XRD pattern of Ga-10% FPA(100 mM) composites, where perpendicular pink lines represent the standard diffraction peaks. This indicated that Ag shell of FPA could indeed react with gallium to produce Ag_xGa_y IMCs in the absence of indium. Source data are provided as a Source Data file.

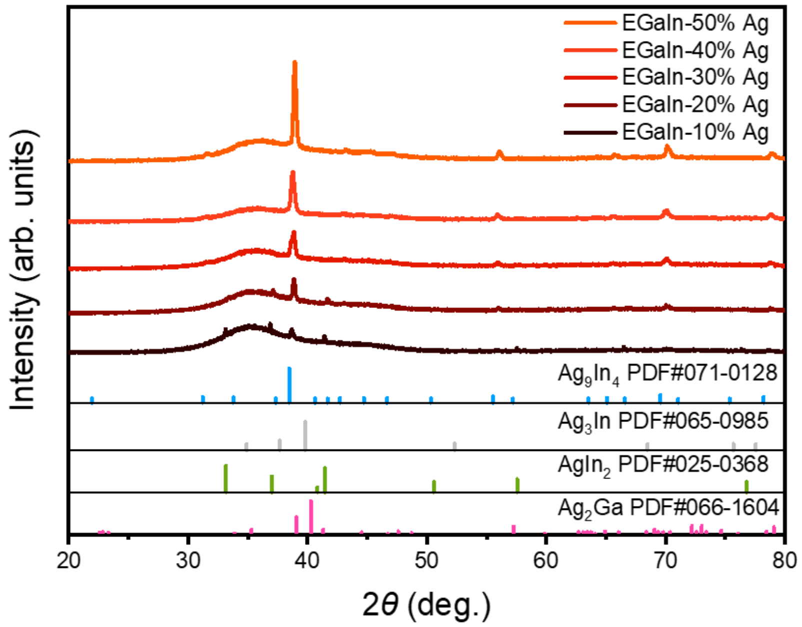

Fig. S18. X-ray patterns of composites with EGaIn and various mass fraction Ag, where standard PDF cards of Ag_9In_4, Ag_3In, $AgIn_2$ and Ag_2Ga are provided for comparison. With the increase of Ag particle amount, the formed IMC phase in liquid metal composite gradually changed from $AgIn_2$ to Ag_9In_4. While Ag_xGa_y IMC did not occur even when the mass ratio between Ag and EGaIn was as high as 50%. Source data are provided as a Source Data file.

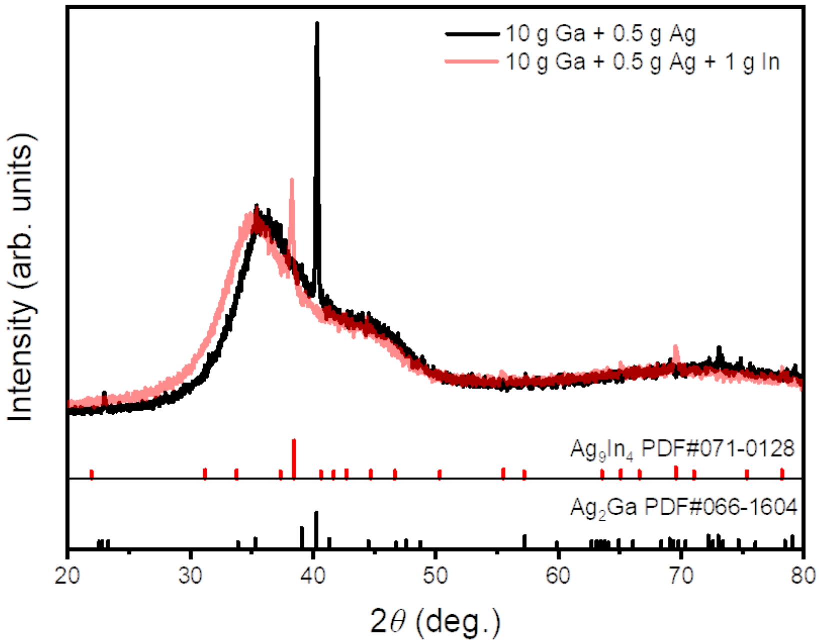

Fig. S19. X-ray patterns of Ag-Ga composite before and after In addition. Ag_2Ga phase was initially identified in Ga-Ag mixture by XRD characteristic peaks. However, Ag_2Ga peaks disappeared after In powder was added, while new peaks corresponding to Ag_9In_4 phase appeared instead, indicating Ag atoms were more inclined to bind with In atoms compared to Ga atoms. Source data are provided as a Source Data file.

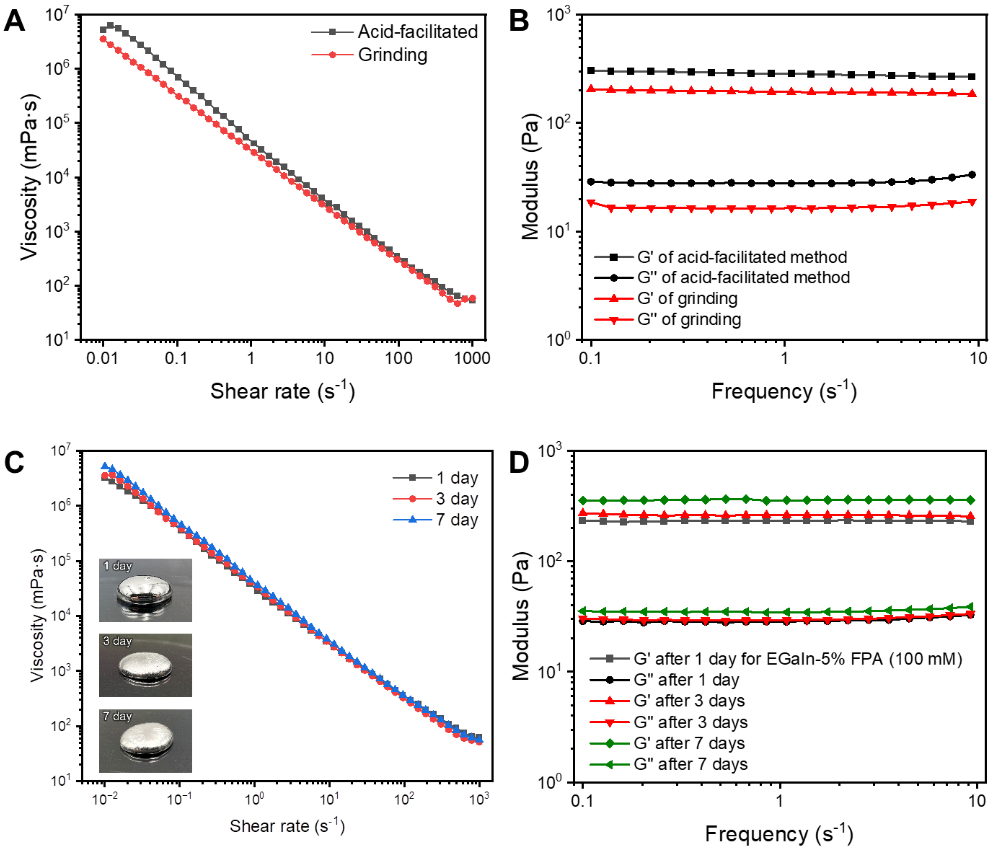

Fig. S20. Rheological properties of magnetic liquid metal composites. (**A**) The viscosity of EGaIn-5% FPA (100 mM) prepared by mechanical grinding and acid-facilitated methods as a function of various shear rates. (**B**) The dynamic storage and loss modulus of EGaIn-5% FPA (100 mM) prepared by mechanical grinding and acid-facilitated methods as a function of various oscillation frequencies, where the strain amplitude was controlled to 5%. It was noted that the viscosity, storage modulus (G'), and loss modulus (G'') of EGaIn-5% FPA (100 mM) prepared by mechanical grinding were slightly lower than those of liquid metal composite prepared by acid-facilitated method. This might be due to emergence of pores and acid corrosion products on the surface of liquid metal composite during the acid-facilitated mixing process. (**C**) The viscosity of EGaIn-5% FPA (100 mM) composites as a function of various shear rates after different periods. (**D**) The dynamic storage modulus and loss modulus of EGaIn-5% FPA (100 mM) composites as a function of various oscillation frequencies after different periods. The phase of liquid metal composite was always at liquid state. Both the viscosity and moduli (storage modulus G' and loss modulus G'') kept relatively stable in the first 3 days, and then slightly increased after 7 days. Therefore, storage of EGaIn-5% FPA (100 mM) liquid metal composite in one week did not significantly affect its rheological property. Source data are provided as a Source Data file.

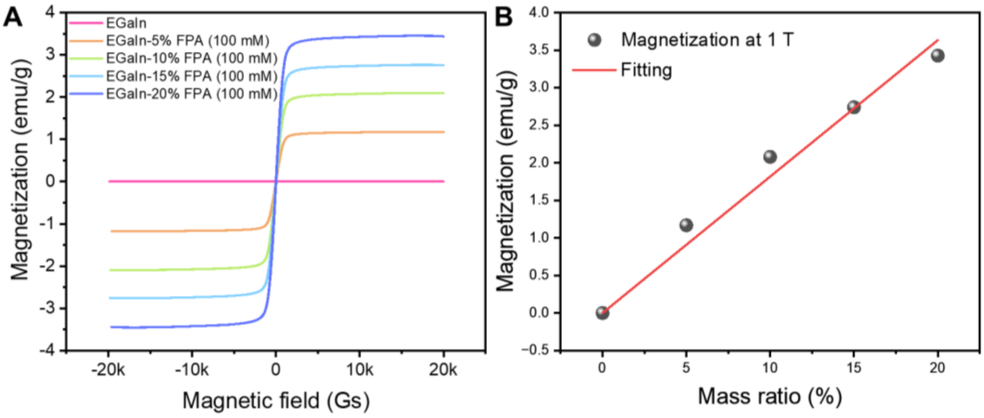

Fig. S21. Magnetic property of pure EGaIn and EGaIn-FPA (100 mM) with various FPA (100 mM) mass ratios. (**A**) The hysteresis loops of EGaIn, EGaIn-5% FPA (100 mM), EGaIn-10% FPA (100 mM), EGaIn-15% FPA (100 mM), and EGaIn-20% FPA (100 mM), showing all of them are paramagnetic due to the magnetism of embedded Fe_3O_4 nanoparticles. (**B**) The effect of FPA mass ratio on the magnetization of liquid metal composite at 1 T, indicating the saturated magnetization is linear correlated positively with the FPA mass ratio. Source data are provided as a Source Data file.

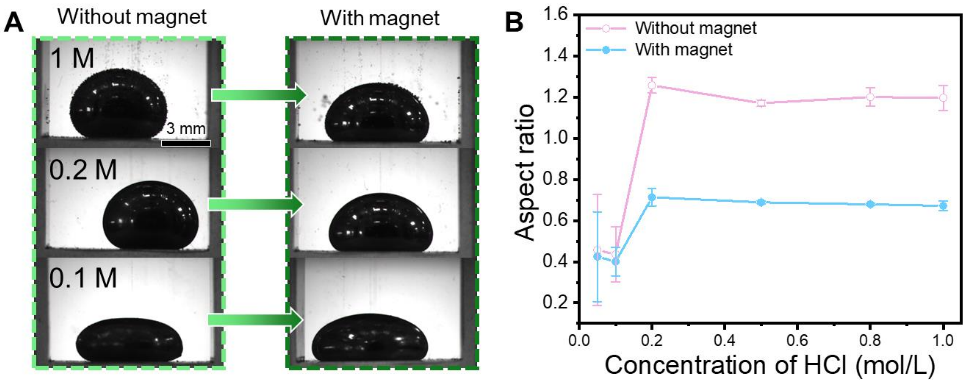

Fig. S22. Effect of HCl solution concentration on the deformation of LMMSR. (**A**) Optical images of LMMSR in different HCl solutions without and with magnets. (**B**) Aspect ratios of LMMSR measured from various concentrations of HCl showing effective deformation can be achieved under external magnetic field, which is nearly consistent with the previous data [6]. All values represent the mean ± SD for n = 3 independent experiments. Source data are provided as a Source Data file.

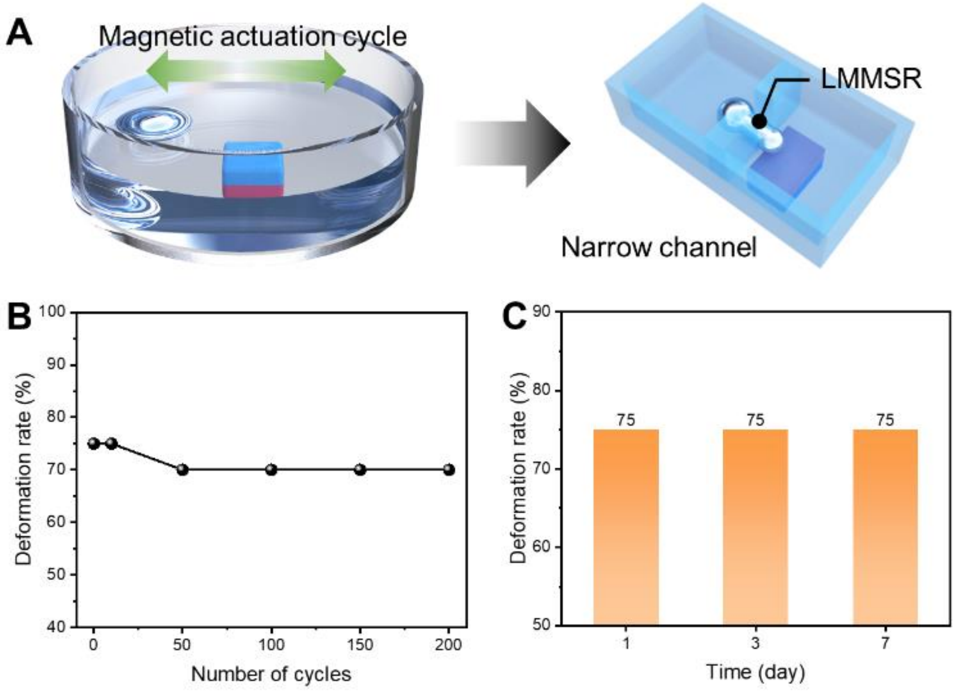

Fig. S23. Stability of LMMSR after actuation. (**A**) Illustrations showing magnetic actuation cycle and the follow-up deformation performance test for LMMSR. We used a permanent magnet to repeatedly drive EGaIn-5% FPA (100 mM) liquid metal composite-based LMMSR along a distance of 60 mm back and forth with a speed of 10 cm/s in hydrochloric acid solution (1 M). After a certain number of cycles (10, 50, 100, 150, and 200), the composite was transferred to a new container to traverse a narrow slit via passive deformation under the guidance of a permanent magnet (surface magnetic field ~500 mT). (**B**) Passive deformation performance of LMMSR as a function of number of cycles. The deformation rate decreased from 75% to 70% after 100 cycles, but the performance remained at 70% as the number of cycles increased. These results indicated that the magnetic actuation performance of LMMSR kept relatively stable and reliable after hundreds of actuation cycles. (**C**) Passive deformation performance of LMMSR as a function of stroge time. The storage time did not affect the deformation ability of liquid metal composite, which might be attributed to the limited change of viscosity. Source data are provided as a Source Data file.

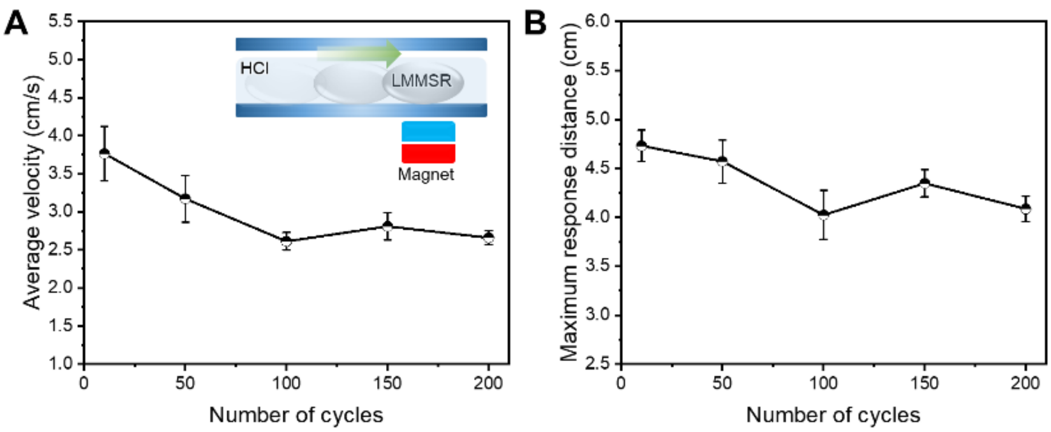

Fig. S24. Stability in magnetic responsiveness after actuation. (**A**) Average velocity of LMMSR as a function of number of cycles where the insert shows the magnetic actuation test after magnetic actuation cycle. (**B**) Maximum response distance as a function of number of cycles. We used a permanent magnet to repeatedly drive EGaIn-5% FPA (100 mM) liquid metal composite-based LMMSR along a distance of 60 mm back and forth with a speed of 10 cm/s in hydrochloric acid solution (1 M). After a certain number of cycles (10, 50, 100, 150, and 200), LMMSR was transferred to a straight tube, and a permanent magnet (surface magnetic field ~500 mT) below tube was controlled to gradually approach LMMSR. When the distance between LMMSR and magnet reached a critical value, the magnetic attractive force would become strong enough to drive LMMSR towards magnet. Such value was defined as maximum response distance of LMMSR, and the average driving velocity of LMMSR towards magnet was also recorded, both of which could demonstrate the magnetic responsiveness of LMMSR. Although the maximum response distance and average velocity of LMMSR decreased after 100 actuation cycles due to the leakage of magnetic agents, the values still remained more than 70% of the original performances when cycle number reached 200. All values represent the mean ± SD for n = 3 independent experiments. Source data are provided as a Source Data file.

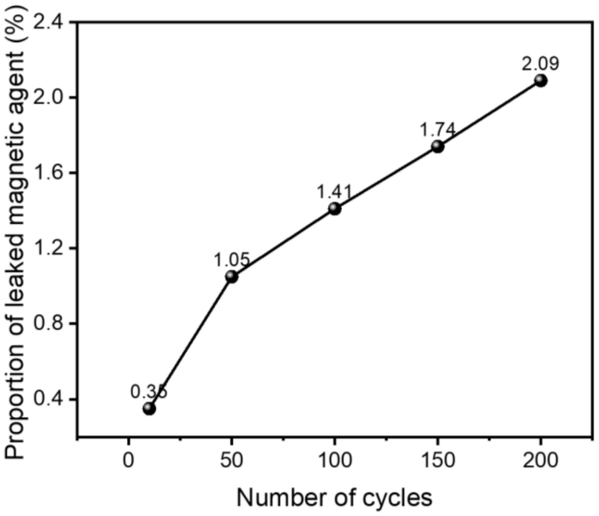

Fig. S25. Mass proportion of leaked magnetic agent as a function of the number of magnetic actuation cycles. We used a permanent magnet to repeatedly drive magnetic liquid metal composite-based LMMSR along a distance of 60 mm back and forth with a speed of 10 cm/s in hydrochloric acid solution (1 M). After a certain number of cycles (10, 50, 100, 150, and 200), the leaked magnetic solids were collected and dissolved in aqua regia, whose content was further measured with ICP-OES. Then the mass proportion of leaked magnetic agents was calculated, which was found to be extremely limited with the increase of cycles. Only 2.09% magnetic nanoparticles leaked out after 200 actuation cycles. Source data are provided as a Source Data file.

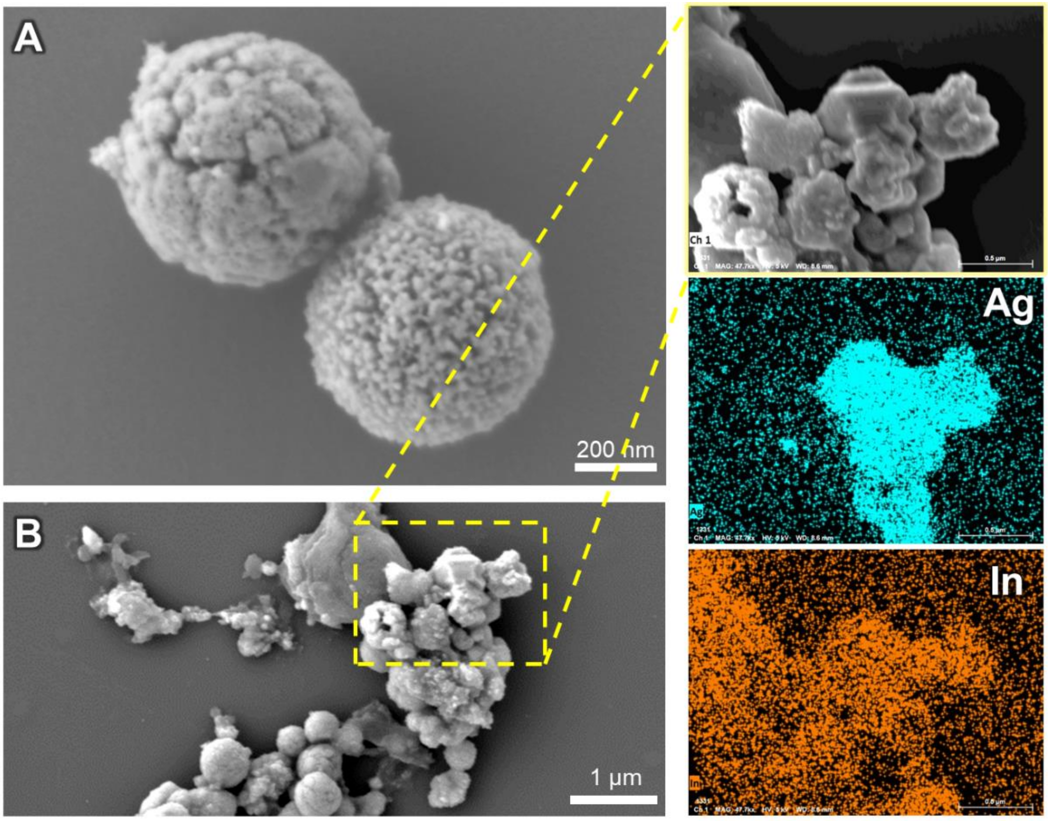

Fig. S26. Characterization of the separated magnetic solids after different actuation cycles. (**A**) SEM image of the leaked magnetic agent. (**B**) SEM image and EDX mappings showing the appearance of leaked magnetic agents with a surface coating of Ag_xIn_y IMC. Each experiment was repeated independently for 3 times with similar results.

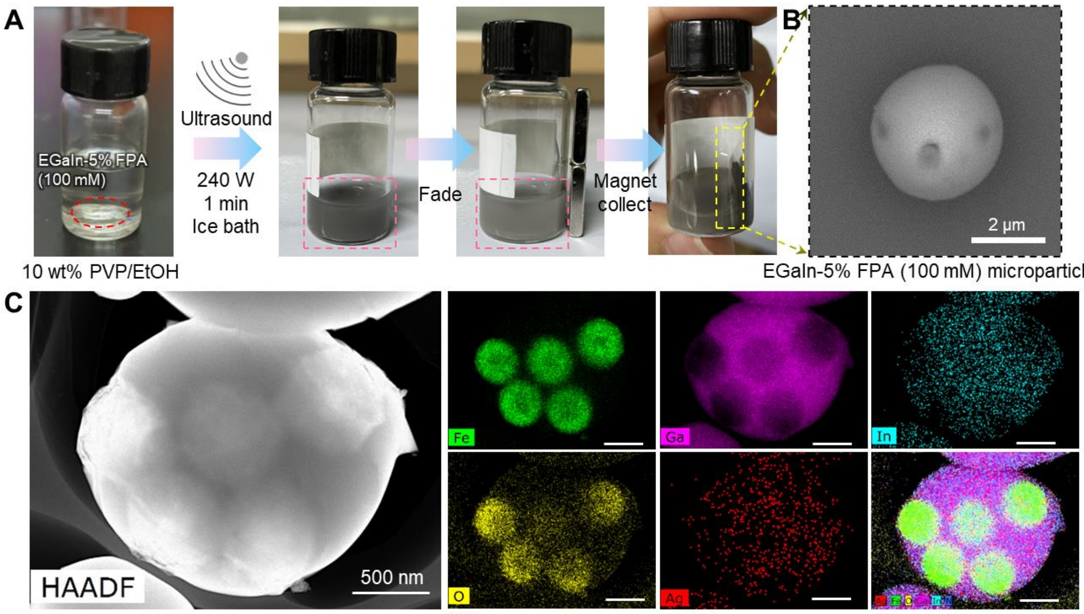

Fig. S27. Preparation and characterization for EGaIn-5% FPA (100 mM) microparticles. (**A**) The preparation process of EGaIn-5% FPA (100 mM) microparticles. EGaIn-5% FPA (100 mM) composites immersed in 10 wt% PVP/EtOH solutions are sheared and broken by probe ultrasound to prepare magnetic liquid metal ink containing EGaIn-5% FPA (100 mM) microparticles. (**B**) SEM image of EGaIn-5% FPA (100 mM) microparticle. (**C**) High-angle annular dark field image and corresponding mappings of EGaIn-5% FPA (100 mM) microparticle where monodispersed Fe_3O_4 nanoparticles are wrapped by EGaIn microparticles, indicating that FPA particles wet with the EGaIn matrix due to the reactive wetting between Ag and In. Moreover, the anchoring effect prevents the separation between Fe_3O_4 and EGaIn. Each experiment was repeated independently for 3 times with similar results.

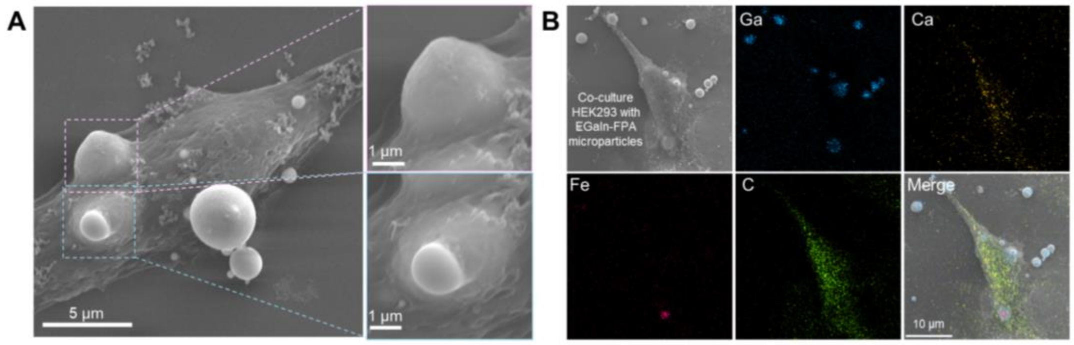

Fig. S28. Co-culture of HEK293 cells with EGaIn-5% FPA (20 mM) microparticles. (**A**) SEM images of HEK293 cell treated by EGaIn-5% FPA (20 mM) microparticles. The magnification of pink dotted area shows that EGaIn-5% FPA (20 mM) microparticle has been endocytosed by the HKE293 cell. The magnification of blue dotted area shows that EGaIn-5% FPA (20 mM) microparticle is semi-endocytosed by the HKE293 cell. Each experiment was repeated independently for 2 times with similar results. (**B**) SEM image and corresponding mappings of HEK293 cell cultured with EGaIn-5% FPA (20 mM) microparticles.

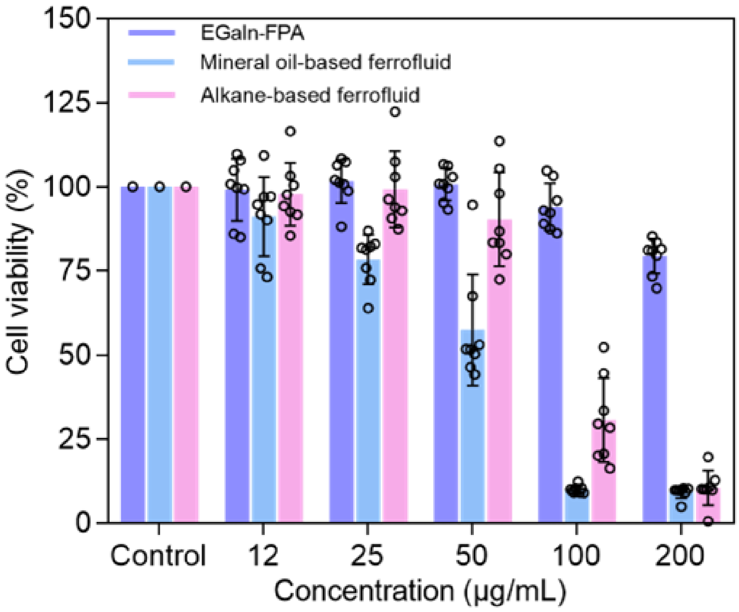

Fig. S29. Cell viability of HEK293 cells cultured with EGaIn-FPA magnetic LM composite, mineral oil-based ferrofluid, and alkane-based ferrofluid at various concentrations for 24 h. When the concentration of magnetic material was as low as 12 μg/mL, all the tested fluids were not harmful to HEK293 cells. However, with the increase of concentration, the cell viabilities experienced a sharp decrease for mineral oil-based and alkane-based ferrofluids. In contrast, more than 80% of cells were still alive when they were incubated with magnetic EGaIn LM composite even with a high concentration of 200 μg/mL. Therefore, our developed LM-based magnetic composite exhibits superior biocompatibility than mineral oil-based and alkane-based ferrofluids. All values represent the mean ± SD for n = 8 independent experiments. Source data are provided as a Source Data file.

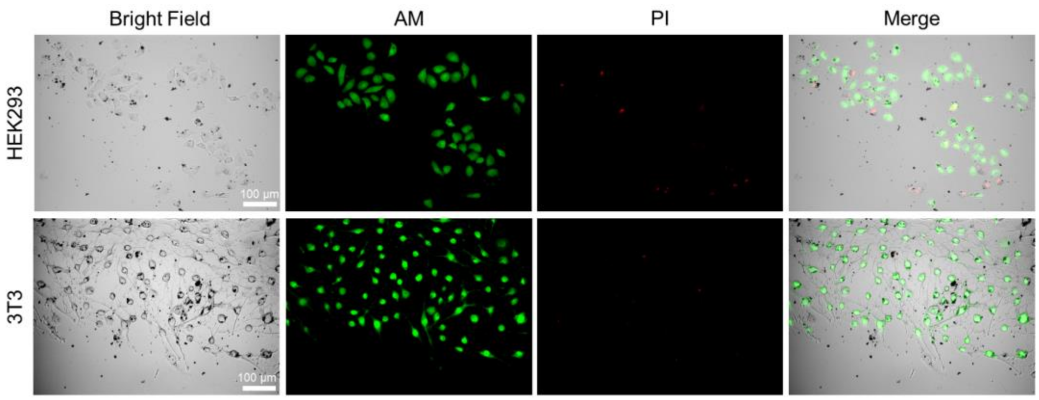

Fig. S30. Bright field and fluorescence images of HEK293 and 3T3 cells stained with Calcein-AM/PI for live/dead test with the addition of EGaIn-5% FPA (20 mM) microparticles (200 μg/mL) for 24 h.

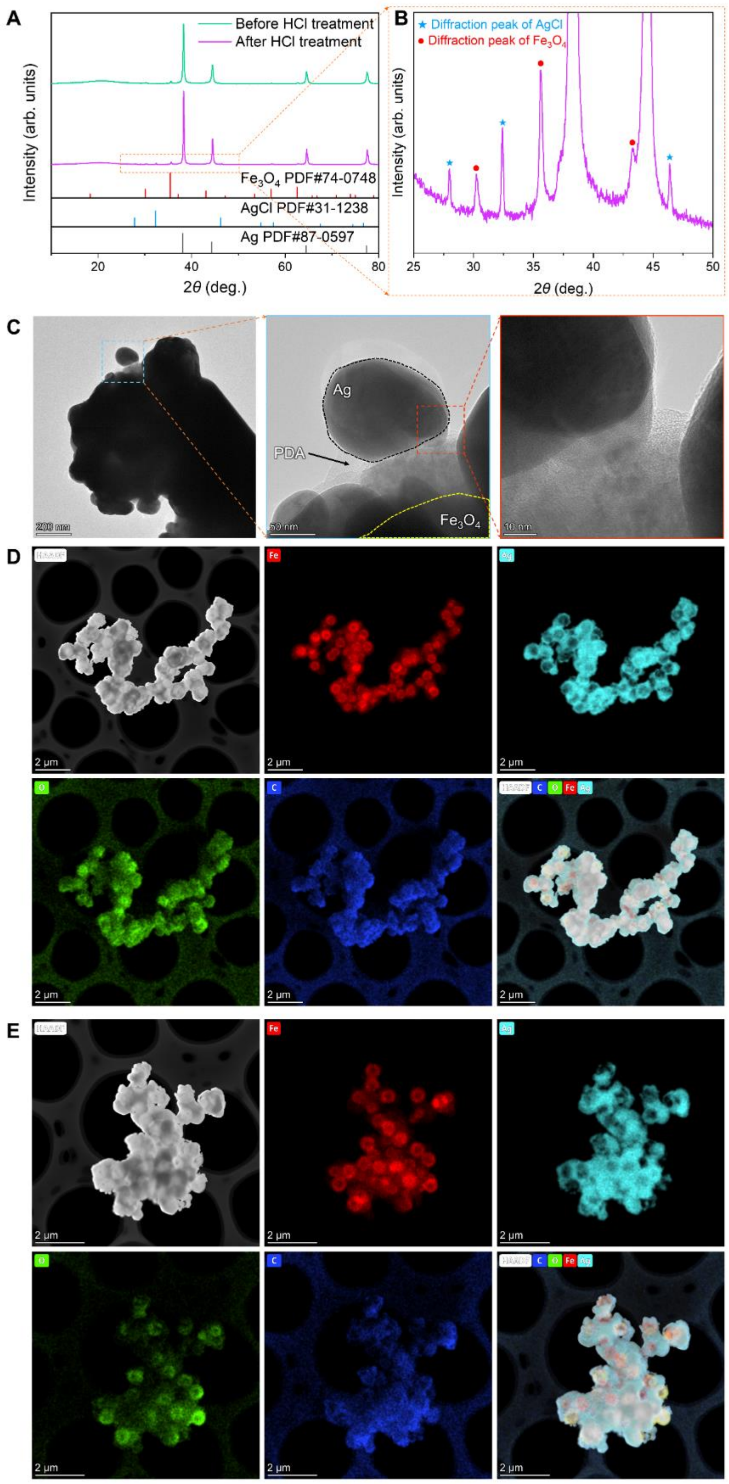

Fig. S31. Stability of FPA nanoparticles in simulated gastric acid. (**A**) XRD for FPA nanoparticles before and after simulated gastric acid treatment and characteristic peaks for Fe_3O_4, AgCl, and Ag, respectively. (**B**) Magnified XRD pattern of FPA nanoparticles after acid treatment. (**C**) TEM images for FPA nanoparticles after acid treatment. (**D**) TEM image and EDX mappings of FPA nanoparticles before acid treatment. (**E**) TEM image and EDX mappings of FPA nanoparticles after acid treatment. All the results verify that FPA nanoparticles are relatively inert with simulated gastric acid, paving the foundation for next-step biomedical applications in stomach. Each experiment was repeated independently for 3 times with similar results. Source data are provided as a Source Data file.

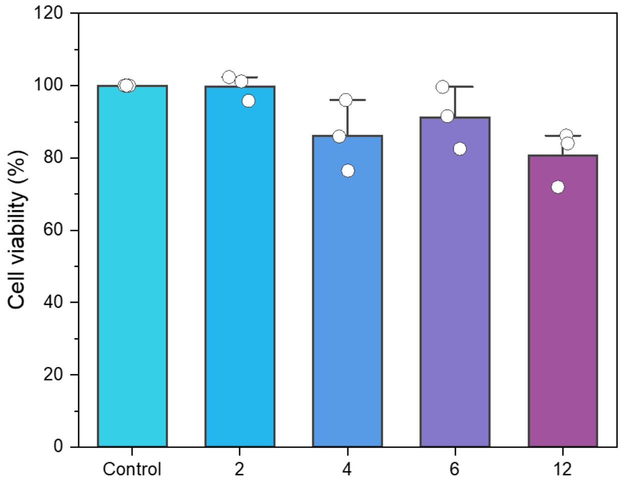

Fig. S32. Viability of HEK293 cells co-cultured with the medium containing Ga (17.40 mg/L), In (1.90 mg/L), Fe (33.52 mg/L) and Ag (3.94 mg/L). All values represent the mean ± SD for n = 3 independent experiments. Source data are provided as a Source Data file.

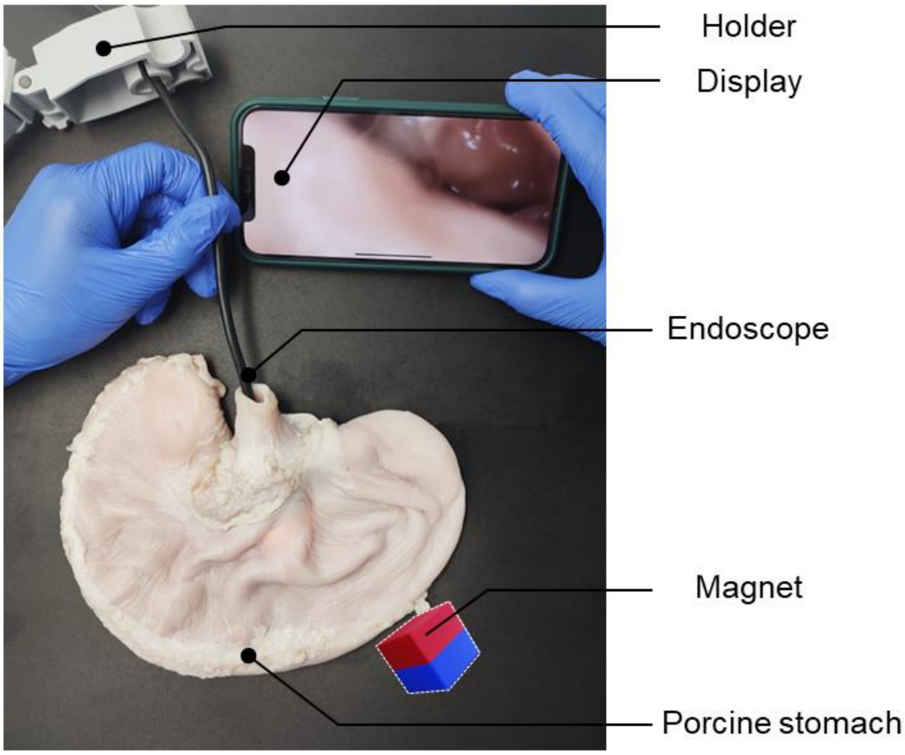

Fig. S33. Magnetic manipulation observed by an endoscope. The holder is used to fix the endoscope into the porcine stomach. The display can observe the manipulation process from endoscope via Bluetooth. In the real experiment, the ex vivo stomach is hung on cantilevers for the ease of magnetic manipulation.

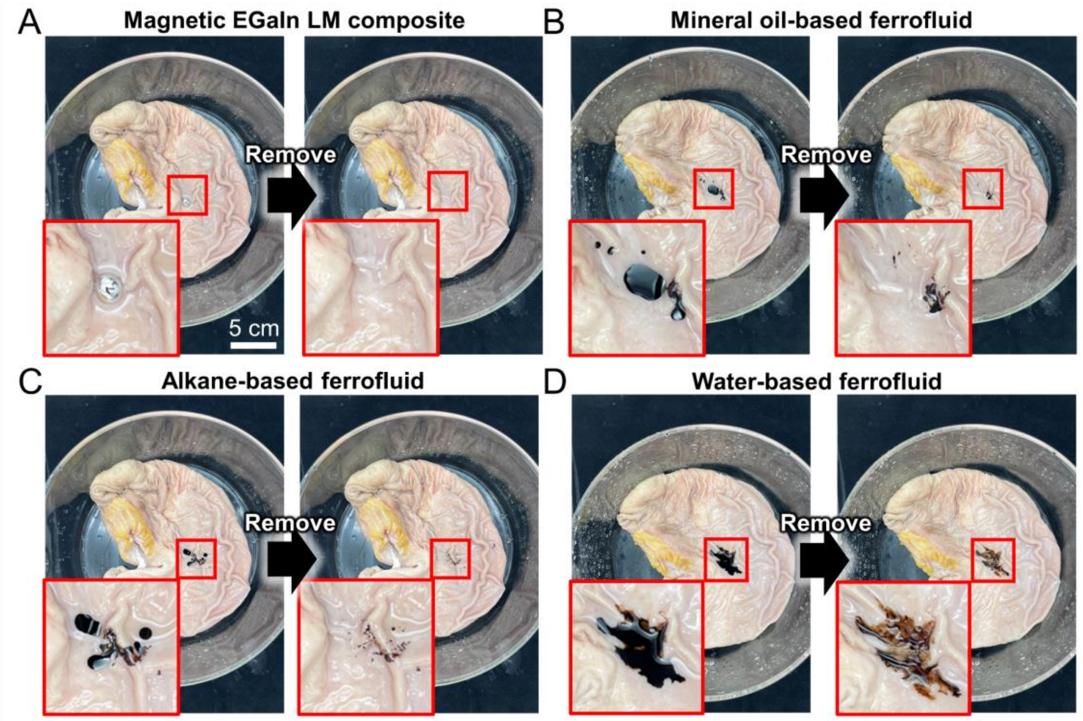

Fig. S34. Optical images showing the appearance of stomach after addition (left) and removal (right) of EGaIn-FPA magnetic LM composite (**A**), mineral oil-based ferrofluid (**B**), alkane-based ferrofluid (**C**), and water-based ferrofluid (**D**). The insets showed the magnified areas surrounded by red boxes. It could be found that magnetic EGaIn LM composite did not adhere to the stomach wall and left no residual material upon removal. In contrast, both mineral oil-based and alkane-based ferrofluids adhered to the stomach wall due to their polar similarity to gastric mucosa, and the corresponding residual materials could not be removed even being flushed by phosphate buffer saline. As to the water-based ferrofluid, it rapidly diffused into the simulated gastric acid upon addition, which could not be actuated by magnetic field or removed by a pipette. Therefore, compared to the commercially available mineral oil-based, alkane-based, and water-based ferrofluids, liquid metal-based magnetic composite exhibits superior immiscibility, which benefits the implementation of biomedical applications.

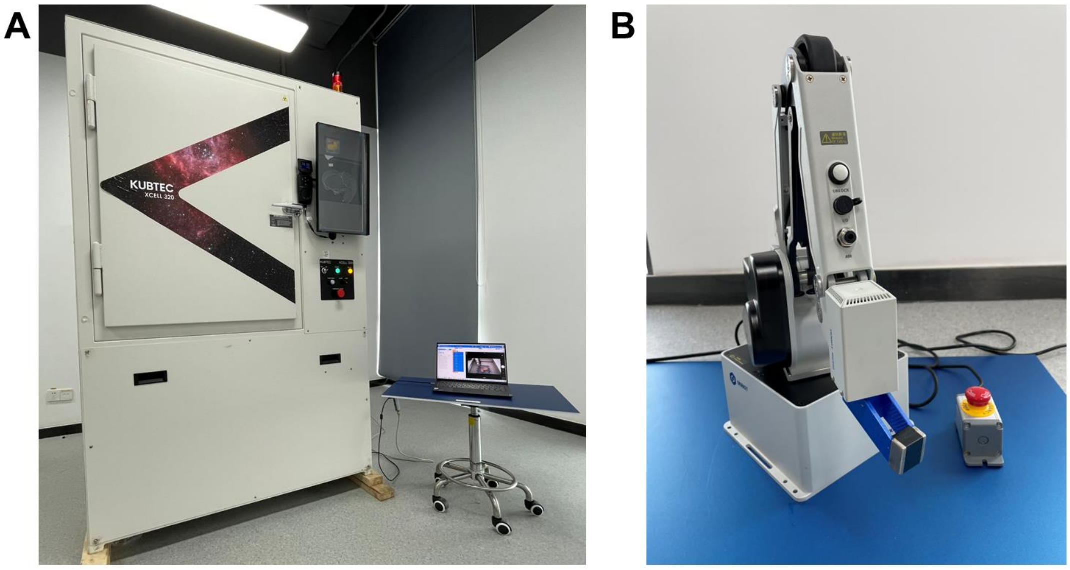

Fig. S35. Equipment for X ray imaging and magnetic manipulation. (**A**) The X ray imaging system and computer during magnetic control of LMMSR in an ex vivo porcine stomach. (**B**) Optical image of a robotic arm integrated with a permanent magnet. The computer can be used to real-time set the position and posture of robotic arm according to the feedback from X ray image.

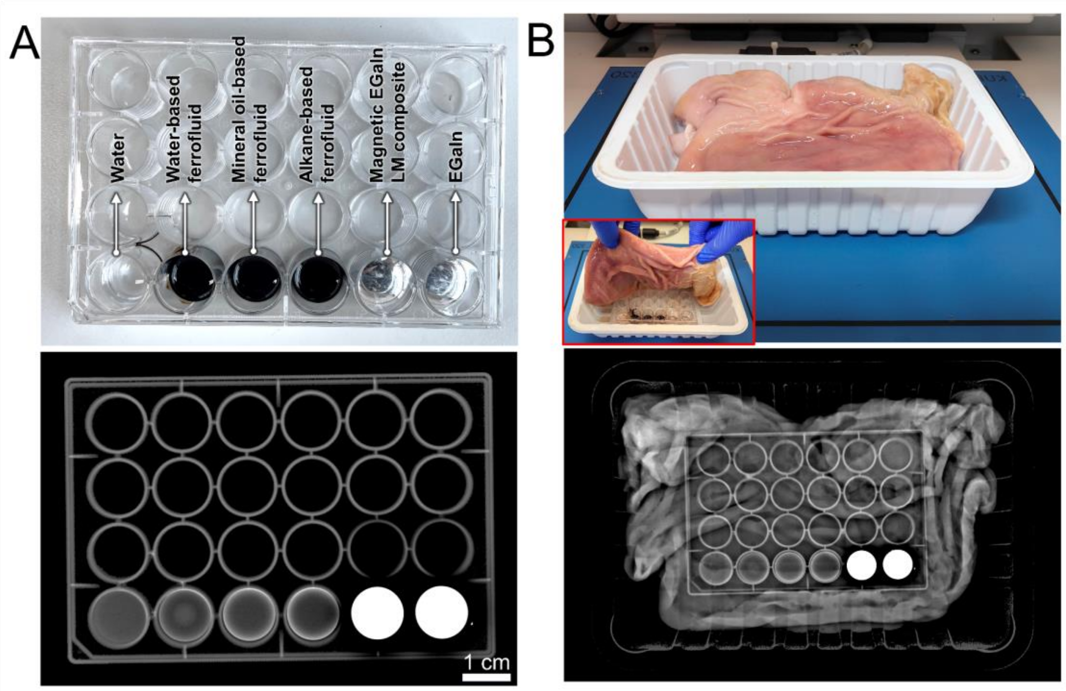

Fig. S36. Comparision between different ferrofluids in X ray imaging contrast. (**A**) Optical image and CT image for various fluids. (**B**) Optical image and CT image for various fluids after covering porcine stomach. Six kinds of liquid, including pure water, water-based ferrofluid, mineral oil-based ferrofluid, alkane-based ferrofluid, magnetic EGaIn LM composite, and pure EGaIn LM, were added to a 24-well plate and imaged using X ray. The fluids containing LM possessed much stronger image contrast compared to other fluids. Even when a fresh porcine stomach was placed over the 24-well plate, the groups containing LM still distinguished themselves from the biological tissues, while the other fluids were not visible compared to background signal. Therefore, LM-based magnetic composite possesses better biomedical imaging performance for promising in vivo applications than other conventional ferrofluids. Each experiment was repeated independently for 3 times with similar results.

Supplementary references

1 Mohammed, M., Sundaresan, R. & Dickey, M. D. Self-Running Liquid Metal Drops that Delaminate Metal Films at Record Velocities. *ACS Appl Mater Interfaces* **7**, 23163-23171, (2015).

2 Shu, J. *et al.* Particle-Based Porous Materials for the Rapid and Spontaneous Diffusion of Liquid Metals. *ACS Appl Mater Interfaces* **12**, 11163-11170, (2020).

3 Wang, H. *et al.* A Liquid Gripper Based on Phase Transitional Metallic Ferrofluid. *Advanced Functional Materials* **31**, (2021).

4 Wu, D. *et al.* A Universal Mechanochemistry Allows On-Demand Synthesis of Stable and Processable Liquid Metal Composites. *Small Methods* **6**, e2200246, (2022).

5 Wang, C. *et al.* A general approach to composites containing nonmetallic fillers and liquid gallium. *Science Advances* **7**, eabe3767, (2021).

6 Xu, Q., Oudalov, N., Guo, Q., Jaeger, H. M. & Brown, E. Effect of oxidation on the mechanical properties of liquid gallium and eutectic gallium-indium. *Physics of Fluids* **24**, (2012).
