# Supplementary material for: Reactive wetting enabled anchoring of non-wettable iron oxide in liquid metal for miniature soft robot
Source: Nat Commun. 2023 Oct 7;14:6276. doi: 10.1038/s41467-023-41920-4 (PMC10560245; doi:10.1038/s41467-023-41920-4)
Supplement: Supplementary file 3 — Description of Additional Supplementary Files [file 41467_2023_41920_MOESM3_ESM.docx]

Supplementary Movie 1.

Description: The extension and contraction of EGaIn droplet on substrates with different FPA particles.

Supplementary Movie 2.

Description: Comparison of preparation process for EGaIn-FPA (100 mM) and EGaIn-Fe_3_O_4_ by mechanical grinding method.

Supplementary Movie 3.

Description: Combination between EGaIn and FPA (100 mM) by electrochemical and acid-facilitated method.

Supplementary Movie 4.

Description: 3D micro-CT demonstration of Ag*_x_*In*_y_* (the green phase) in EGaIn-5% FPA (100 mM) magnetic liquid metal composite.

Supplementary Movie 5.

Description: Shape switching and reconfiguration for LMMSR.

Supplementary Movie 6.

Description: On-demand manipulation for LMMSR under magnetic field.

Supplementary Movie 7.

Description: Locomotion and fusion for sub-LMMSR.

Supplementary Movie 8.

Description: Obstacle crossing, cargo handling, splitting, and homing for LMMSR under magnetic control in channel.

Supplementary Movie 9.

Description: Manipulation for LMMSR in an ex vivo porcine stomach under the actuation of magnetic field.

Supplementary Movie 10.

Description: X-ray imaging for LMMSR in an ex vivo porcine stomach.
