# Supplementary material for: Reactive wetting enabled anchoring of non-wettable iron oxide in liquid metal for miniature soft robot
Source: Nat Commun. 2023 Oct 7;14:6276. doi: 10.1038/s41467-023-41920-4 (PMC10560245; doi:10.1038/s41467-023-41920-4)
Supplement: Supplementary file 14 — Reporting Summary [file 41467_2023_41920_MOESM14_ESM.pdf]

nature portfolio

Corresponding author(s): Dongdong Jin, Xing Ma

Last updated by author(s): Sep 9, 2023

Reporting Summary

Nature Portfolio wishes to improve the reproducibility of the work that we publish. This form provides structure for consistency and transparency in reporting. For further information on Nature Portfolio policies, see our Editorial Policies and the Editorial Policy Checklist.

Statistics

For all statistical analyses, confirm that the following items are present in the figure legend, table legend, main text, or Methods section.

n/a	Confirmed	
	x	The exact sample size (n) for each experimental group/condition, given as a discrete number and unit of measurement
	x	A statement on whether measurements were taken from distinct samples or whether the same sample was measured repeatedly
x		The statistical test(s) used AND whether they are one- or two-sided<br>*Only common tests should be described solely by name; describe more complex techniques in the Methods section.*
	x	A description of all covariates tested
x		A description of any assumptions or corrections, such as tests of normality and adjustment for multiple comparisons
	x	A full description of the statistical parameters including central tendency (e.g. means) or other basic estimates (e.g. regression coefficient) AND variation (e.g. standard deviation) or associated estimates of uncertainty (e.g. confidence intervals)
x		For null hypothesis testing, the test statistic (e.g. F, t, r) with confidence intervals, effect sizes, degrees of freedom and P value noted<br>*Give P values as exact values whenever suitable.*
x		For Bayesian analysis, information on the choice of priors and Markov chain Monte Carlo settings
x		For hierarchical and complex designs, identification of the appropriate level for tests and full reporting of outcomes
x		Estimates of effect sizes (e.g. Cohen's d, Pearson's r), indicating how they were calculated

Our web collection on statistics for biologists contains articles on many of the points above.

Software and code

Policy information about availability of computer code

Data collection	The data were collected on X-ray diffractometer (D Max-2500 Pc), Fourier-transformed infrared spectrometer (Nicolet380), dynamic light scattering (Zetasizer Nano ZEP), scanning electron microscopy (ProX), Leica inverted optical microscope (DMi8), transmission electron microscopy (Talos F200x), rheometer (Anton Paar MCR72), vibrating sample magnetometer (LakeShore7404), contact angle meter (JC2000D), three-dimensional X-ray microscopy (Zeiss Versa 515), inductively coupled plasma optical emission spectra (PerkinElmer Avio 200), microplate reader (Infinite F50 Tecan), and XCELL 320 irradiator system (Kubtec Scientific).
Data analysis	Origin 2018, Dragonfly, COMSOL Multiphysics 6.1, Graphpad Prism 8

For manuscripts utilizing custom algorithms or software that are central to the research but not yet described in published literature, software must be made available to editors and reviewers. We strongly encourage code deposition in a community repository (e.g. GitHub). See the Nature Portfolio guidelines for submitting code & software for further information.

Data

Policy information about availability of data

All manuscripts must include a data availability statement. This statement should provide the following information, where applicable:

- Accession codes, unique identifiers, or web links for publicly available datasets
- A description of any restrictions on data availability
- For clinical datasets or third party data, please ensure that the statement adheres to our policy

The authors declare that the data supporting the findings of this study are available within the paper and its supplementary information files. Source data are

provided with this paper.

Research involving human participants, their data, or biological material

Policy information about studies with human participants or human data. See also policy information about sex, gender (identity/presentation), and sexual orientation and race, ethnicity and racism.

Reporting on sex and gender	this information has not been collected
Reporting on race, ethnicity, or other socially relevant groupings	this information has not been collected
Population characteristics	not applicable
Recruitment	not applicable
Ethics oversight	not applicable

Note that full information on the approval of the study protocol must also be provided in the manuscript.

Field-specific reporting

Please select the one below that is the best fit for your research. If you are not sure, read the appropriate sections before making your selection.

☒ Life sciences ☐ Behavioural & social sciences ☐ Ecological, evolutionary & environmental sciences

For a reference copy of the document with all sections, see nature.com/documents/nr-reporting-summary-flat.pdf

Life sciences study design

All studies must disclose on these points even when the disclosure is negative.

Sample size	In this work, the aim is to verify the function of liquid metal soft robot in an ex vivo porcine stomach. The sample size has no effect on the conclusion.
Data exclusions	No data were excluded from the analyses.
Replication	All experiments were repeated a minimum of one time to confirm the repeatability of the findings. All repeats of experiments were successful.
Randomization	Random grouping.
Blinding	No formal blinding was used for no assessments were necessary.

Reporting for specific materials, systems and methods

We require information from authors about some types of materials, experimental systems and methods used in many studies. Here, indicate whether each material, system or method listed is relevant to your study. If you are not sure if a list item applies to your research, read the appropriate section before selecting a response.

Materials & experimental systems

n/a	Involved in the study	
☒	☐	Antibodies
☐	☒	Eukaryotic cell lines
☒	☐	Palaeontology and archaeology
☒	☐	Animals and other organisms
☒	☐	Clinical data
☒	☐	Dual use research of concern
☒	☐	Plants

Methods

n/a	Involved in the study	
☒	☐	ChIP-seq
☒	☐	Flow cytometry
☒	☐	MRI-based neuroimaging

Eukaryotic cell lines

Policy information about cell lines and Sex and Gender in Research

Cell line source(s)	human embryonic kidney HEK293 cells and mouse embryonic fibroblasts 3T3 cells were purchased from BeNa Culture Collection, China
Authentication	each cell line used was authenticated by the supplier via morphology
Mycoplasma contamination	all cell lines were tested negative for mycoplasma contamination
Commonly misidentified lines (See ICLAC register)	No
